# Diverse modes of ceftazidime/avibactam resistance acquisition in carbapenem-resistant *Klebsiella pneumoniae* and *Pseudomonas aeruginosa* from a Chinese intensive care unit

**DOI:** 10.1186/s12941-025-00800-z

**Published:** 2025-05-30

**Authors:** Junxin Zhou, Minhua Chen, Min Liang, Xinhong Han, Rui Weng, Yue Li, Yan Jiang, Xiaoting Hua, Xiaoxing Du, Weiping Wang, Zhihui Zhou, Yunsong Yu

**Affiliations:** 1 Department of Infectious Diseases, Sir Run Run Shaw Hospital, Zhejiang University School of Medicine, No. 3, East Qingchun Rd, Shangcheng District, Hangzhou, 310016 China; 2 Key Laboratory of Microbial Technology and Bioinformatics of Zhejiang Province, Hangzhou, China; 3 Regional Medical Center for National Institute of Respiratory Diseases, Sir Run Run Shaw Hospital, Zhejiang University School of Medicine, Hangzhou, China; 4 Department of Clinical Laboratory, Zhejiang Cancer Hospital, Hangzhou, Zhejiang China; 5 Department of Clinical Laboratory, Jinling Hospital, Medical School of Nanjing University, Nanjing, China

**Keywords:** *Pseudomonas aeruginosa*, *Klebsiella pneumoniae*, Ceftazidime/avibactam, KPC, Non-Tn*4401* elements, Cross-regional transmission

## Abstract

**Objectives:**

To investigate the mechanisms of ceftazidime/avibactam (CZA) resistance and the nosocomial dissemination of carbapenem-resistant *Pseudomonas aeruginosa* (CRPA) and carbapenem-resistant *Klebsiella pneumoniae* (CRKP) in an intensive care unit (ICU) in China.

**Methods:**

Clinical CRPA and CRKP isolates were obtained from an ICU of a tertiary hospital in China from August 2020 to February 2021. Antimicrobial susceptibility was determined according to CLSI. WGS, cloning experiments and kinetic parameters were conducted to reveal resistance mechanisms, molecular characteristics and dissemination of CRPA and CRKP.

**Results:**

We isolated 32 CZA-resistant strains, including 12 CRPA and 20 CRKP strains from an ICU between August 2020 and February 2021. CZA resistance was associated with the presence of NDM and efflux pumps in CRKP strains, whereas *bla*_AFM-2_, *bla*_KPC-87_, and *bla*_PER-1_ contributed to CZA resistance in CRPA strains. Compared to KPC-2, KPC-87 exhibited a 1.5-fold elevation in *k*_cat_/*K*_m_ for ceftazidime, a 7.5-fold increase in *K*_i_ for avibactam, and a loss of carbapenem hydrolysis. *bla*_KPC-87_ was located in the NTE_KPC_-IIa like element based on the Tn*3*. Insertion of 656 bp Δ*bla*_TEM-1_ upstream of *bla*_KPC-87_ introduced an additional promoter that increased KPC-87 expression. Cluster 2 and 3 of CRKP represented two different clones of ST11 transmitted between patients. KPC-87-producing ST270 CRPA strains exhibited a small-scale dissemination and cross-regional transfer with the referral of a patient. The evolutionary pathways of AFM-2-producing ST275 CRPA strains were more complex to elucidate the transmission events.

**Conclusions:**

In CRKP and CRPA, diverse resistance mechanisms contributed to CZA resistance. These CZA-resistant strains were transmitted among patients in the ICU and even across regions to the other healthcare unit when the patient was transferred.

**Supplementary Information:**

The online version contains supplementary material available at 10.1186/s12941-025-00800-z.

## Introduction

CZA, as one of the earlier next-generation β-lactam/β-lactamase inhibitor (BLBLI), has been clinically used for the treatment of complicated intra-abdominal infections and hospital-acquired pneumonia since 2015 [1]. While CZA exhibits potent in vitro activity against many of CRPA and CRKP strains [2, 3], CZA resistance has increased markedly in recent years due to its widespread usage [4]. Emerging KPC variants, identified over the past two years, mediated CZA resistance in through a “seesaw effect” that restores carbapenem susceptibility in CRKP but not in CRPA [4–9]. Notably, Occurrence of KPC variants and metallo-β-lactamases (MBLs) in CRPA, along with MBL-producing CRKP, frequently results in carbapenem-CZA co-resistance, further limiting therapeutic options.

Transmission of carbapenem-resistant Gram-negative bacteria, particularly in ICUs, drives hospital outbreaks due to overcrowding, invasive procedures, and high antibiotic exposure [10]. In China, *bla*_KPC-2_ dominates among CRPA and CRKP isolates, with KPC-producing *P. aeruginosa* (KPC-PA) being endemic to eastern regions such as Zhejiang, Jiangsu and Shanghai [11–14]. Despite importance of CZA in treating these infections, over 50% of KPC-PA isolates in eastern China exhibited CZA resistance [11, 13], underscoring the urgent need to unravel resistance mechanisms and transmission dynamics.

While CRPA nosocomial outbreaks typically originate from hospital-adapted clones, cross-regional transmission of multidrug-resistant *P. aeruginosa* (MDR-PA) is rarely documented. In this study, we observed diverse CZA resistance mechanisms in CRPA and CRKP infecting ICU patients, along with cross-regional dissemination of a CZA-resistant CRPA clone linked to patient referral. This study aimed to [1] elucidate the genetic and molecular mechanisms underlying CZA resistance in CRKP and CRPA, [2] investigate the transmission dynamics of CZA-resistant strains within and beyond the ICU, and [3] identify epidemiological links between clonal dissemination and patient outcomes.

## Materials and methods

### Bacterial isolates and antimicrobial susceptibility testing

12 clinical CZA-resistant CRPA isolates and 20 clinical CZA-resistant CRKP isolates, which were isolated from non-repeated specimens, were collected from an ICU of a tertiary hospital in Jiangsu, China in August 2020-February 2021. Minimum inhibitory concentrations (MICs) were determined according to the Clinical and Laboratory Standards Institute (CLSI) recommendations (M07). *E. coli* ATCC 25922 and *K. pneumoniae* ATCC 700603 served as control strains. Results were interpreted according to CLSI breakpoints except for fosfomycin. For fosfomycin, we interpreted the results based on CLSI breakpoints of *Enterobacterales*. *P. aeruginosa* PA0105 was from our laboratory strain library, which carries *bla*_KPC-2_. The efflux pump inhibition assay was performed by measuring the MICs of CZA with or without 50 mg/L Phe-Arg-β-naphthylamide (PAβN) (Takara Bio Inc., Otsu, Shiga, Japan).

### Whole genome sequencing (WGS) analysis

The genomic DNA of isolates were extracted by QIAamp DNA MiniKit (Qiagen, New York, USA) and subjected to Illumina paired-end sequencing (Illumina Inc., San Diego, CA). De novo assembly of genomes were accomplished by shovill 0.9.0 (https://github.com/tseemann/shovill). Multilocus sequence typing (MLST) and antibiotic resistance genes (ARGs) were recognized with mlst v2.19.0 (https://github.com/tseemann/mlst) and ABRicate v1.0.1 (https://github.com/tseemann/abricate), respectively. *P. aeruginosa* isolates carried *bla*_KPC_ were selected for Nanopore MinION long-read sequencing (Oxford Nanopore Technologies, Oxford, UK). Nanopore long reads of each isolate were hybrid assembled with the corresponding Illumina short reads via Unicycler v.0.4.8 (https://github.com/rrwick/Unicycler). Plasmid size was identified for AFM-producing strains using S1-PFGE [15].

Genomes of all isolates were annotated using Prokka v1.14.5 (https://github.com/tseemann/prokka). The phylogenetic tree was constructed using Panaroo v1.2.9 (https://github.com/gtonkinhill/panaroo), IQ-TREE v2.1.4 (https://github.com/Cibiv/IQ-TREE) and visualized with iTOL (https://itol.embl.de/). Core genome single-nucleotide polymorphisms (cgSNPs) among strains were calculated using Snippy v4.4.5 (https://github.com/tseemann/snippy) and visualized with R package pheatmap v1.0.12 (https://cran.r-project.org/web/packages/pheatmap/). The criteria for classifying CRKP and CRPA as the same clone based on cgSNPs are less than 25 and 26, respectively [10, 16].

*bla*_NDM_ and *bla*_KPC_ copy number were determined by dividing the gene depth by the average depth of seven MLST alleles. And the sequencing depth were obtained by mapping Illumina raw reads to reference sequences of *bla*_NDM_, *bla*_KPC_ and seven MLST alleles using bowtie2 version 2.2.5 [17]. The genetic context comparison was performed and visualized by Easyfig 2.2.5 (http://easyfig.sourceforge.net/). The mapping was processed and visualized by Geneious Prime 2022 (https://www.geneious.com). Mauve alignment of IncP-2 plasmids was also performed by Geneious Prime 2022. Promoter prediction for KPC-87 was performed using the PromoterHunter [18, 19].

### Cloning experiments

Due to differences in the promoter sequences upstream of *bla*_KPC-87_ and *bla*_KPC-2_ and in order to avoid the strong driving effect of the PY promoter masking the potential regulatory role of the Δ*bla*_TEM-1-_ on gene expression, we constructed *bla*_KPC_ plasmids with different promoter (Table 3). The cloned sequences were homologously recombined with the same pGK1900 plasmid backbone. The recombinant plasmids were then transformed into *E. coli* DH5α and *P. aeruginosa* PAO1. Quantitative PCRs were performed to evaluate the expression level of KPCs in PAO1 transformants with five biological repetitions using TB Green Premix ExTaq (TaKaRa, Beijing, China). The transformants of *bla*_PER-1_ were constructed using the same method. The transformants were verified by Sanger sequencing and tested for antimicrobial susceptibility. Primers for cloning experiments and quantitative PCR are listed in Table S1.

### Protein purification and steady-state kinetics assays

As previously described [9], we constructed the expression vectors of KPC-2 and KPC-87. We used *E. coli* Origami2(DE3) as expression strains and protein purification was performed as previously described [15]. We used a spectrophotometer (Rumqee, Shanghai, China) to measure the steady-state kinetics of KPC enzymes in phosphate buffered saline (PBS, pH 7.2) at room temperature. For hydrolysis substrates (nitrocefin and ceftazidime), we fitted the data into the Michaelis–Menten equation to obtain the *K*_m_ and* k*_cat_ by GraphPad Prism v9.4.1. We also performed direct competition assays for avibactam with 100 μM nitrocefin. Generally, KPC enzymes at a fixed concentration were mixed with inhibitors at various concentrations ([I]). 100 mM nitrocefin ([S]) was then added, and the initial reaction velocities (*v*) were measured. *K*i values were obtained by fitting the data into Eq. (1) below using GraphPad Prism v9.0.0. [9, 20].

1 $$v=\frac{{V}_{max}\left[\text{S}\right]}{\left[\text{S}\right]+{K}_{\text{m NCF}}\left(1+\frac{\left[\text{I}\right]}{{K}_{\text{i}}}\right)}$$

Progress curves of KPC variants for meropenem hydrolysis were generated by measuring absorbance at 300 nm with 30 nM enzymes and 50 μM meropenem.

## Results

### Antimicrobial resistance characteristic

**Fig. 1:**
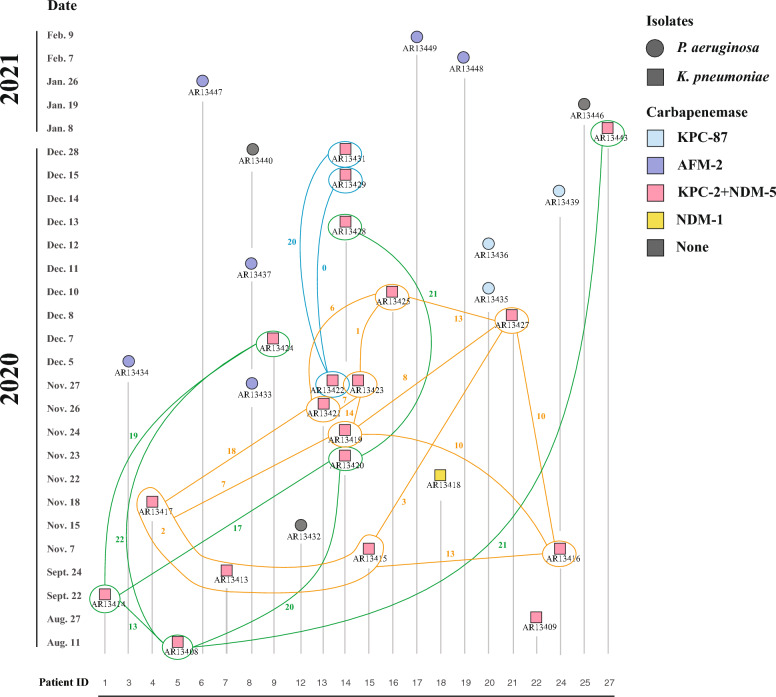
Isolation time and molecular characterization of CZA-resistant strains. Isolation time and source of CZA-resistant strains. Squares represent *K.* pneumoniae strains and circles represent *P. aeruginosa* strains. Different colours represent various carbapenemase-carrying characteristics. The strains in *K. pneumoniae* cluster 1 and SNPs among the strains were marked by blue line. The orange line and the green line marked the strains in *K. pneumoniae* cluster 2 and cluster 3, respectively. Only the two smallest SNPs with similar separation times are labelled

The timeline of isolation of 32 CZA-resistant CRPA and CRKP strains is shown in Fig. 1. All 12 CRPA strains were resistant both meropenem and imipenem (Table 1). Six AFM-2 producing *P. aeruginosa* (AFM-2-PA) strains were highly resistant to carbapenems (MICs > 256 mg/L), while the other 6 CRPA strains including 3 KPC-87 producing *P. aeruginosa* (KPC-87-PA) strains were moderately resistant to carbapenems (MICs = 16–64 mg/L). KPC-87-PA, AFM-2-PA, along with one non-carbapenemase-producing strain exhibited high-level resistance to CZA (MIC ≥ 128 mg/L). In contrast, the remaining two non-carbapenemase-producing strains showed low-level resistance to CZA (MIC = 16–32 128 mg/L). All CRPA strains were resistant to levofloxacin and sensitive to fosfomycin and colistin. AFM-2-PA were highly resistant to meropenem/vaborbactam and imipenem/relebactam while two strains were resistant to aztreonam/avibactam. In addition to meropenem/vaborbactam and imipenem/relebactam, most non-AFM-2-producing strains were also resistant to aztreonam/avibactam.

**Table 1:** Antimicrobial susceptibility of clinical *P. aeruginosa* isolates in this study

Strains	MICs (mg/L)											Carbapenemase	
	IMP	MEM	AMK	LEV	FOS	COL	MEV	IMR	AZA	CZA	CZA^a^	KPC-87	AFM-2
AR13432	**32**	**64**	8	**16**	16	2	**32**	**8**	**64**	**32**	4	**−**	**−**
AR13440	**16**	**16**	8	**32**	16	2	**16**	1	**32**	**16**	0.25	**−**	**−**
AR13446	**16**	**64**	**> 256**	**4**	128	0.5	**32**	**8**	**64**	**128**	2	**−**	**−**
AR13435	**16**	**64**	2	**32**	32	1	**32**	**32**	**64**	**> 128**	**128**	**+**	**−**
AR13436	**16**	**64**	2	**32**	32	2	**64**	**32**	**64**	**> 128**	**128**	**+**	**−**
AR13439	**16**	**64**	2	**32**	32	1	**64**	**32**	**64**	**> 128**	**128**	**+**	**−**
AR13433	**> 256**	**> 256**	**> 256**	**> 128**	32	2	**> 128**	**> 128**	4	**> 128**	**> 128**	**−**	**+**
AR13434	**> 256**	**> 256**	**> 256**	**> 128**	16	2	**> 128**	**> 128**	4	**> 128**	**> 128**	**−**	**+**
AR13437	**> 256**	**> 256**	**64**	**32**	16	2	**> 128**	**> 128**	**32**	**> 128**	**> 128**	**−**	**+**
AR13447	**> 256**	**> 256**	**> 256**	**> 128**	32	1	**> 128**	**> 128**	8	**> 128**	**> 128**	**−**	**+**
AR13448	**> 256**	**> 256**	**> 256**	**> 128**	16	1	**> 128**	**> 128**	8	**> 128**	**> 128**	**−**	**+**
AR13449	**> 256**	**> 256**	**> 256**	**> 128**	16	1	**> 128**	**> 128**	**32**	**> 128**	**> 128**	**−**	**+**

MIC in bold indicates exceeding the resistance breakpoint

^a^The efflux pump inhibition assay was performed by measuring the MICs of CZA with or without 50 mg/L Phe-Arg-β-naphthylamide (PAβN)

*IMP* imipenem, *MEM* meropenem, *AMK* amikacin, *LEV* levofloxacin, *FOS* fosfomycin, *COL* colistin, *MEV* meropenem/vaborbactam, *IMR* imipenem/relebactam, *AZA* aztreonam/avibactam, *CZA* ceftazidime/avibactam, *MIC* minimum inhibitory concentration

In contrast, all CRKP strains displayed the same antimicrobial susceptibility phenotype. Almost all KPC-2 and NDM-5 co-producing CRKP (KPC-2-NDM-5 CRKP) strains were resistant to amikacin, levofloxacin, fosfomycin, ceftazidime/avibactam, meropenem/vaborbactam and imipenem/relebactam but remained sensitive to colistin, tigecycline and aztreonam/avibactam (Table 2). AR13418 producing NDM-1 exhibited a distinct phenotype compared to other strains, as it only showed resistance to meropenem, levofloxacin, tigecycline and ceftazidime/avibactam. All strains were identified as multi-drug resistant strains according to definitions established by Magiorakos et al.[21].

**Table 2:** Antimicrobial susceptibility of clinical *K. pneumoniae* isolates in this study

Strain	MICs (mg/L)											Carbapenemase
	IMP	MEM	AMK	LEV	FOS	COL	MEV	IMR	AZA	CZA	TGC	
AR13408	**64**	**128**	**> 128**	**32**	**256**	0.25	**> 64**	**> 128**	0.25	**> 128**	0.5	KPC-2, NDM-5
AR13409	**128**	**128**	0.5	**32**	16	0.5	**64**	**> 128**	0.5	**> 128**	2	KPC-2, NDM-5
AR13413	**64**	**256**	**> 128**	**> 64**	**512**	0.25	**> 64**	**128**	0.5	**> 128**	4	KPC-2, NDM-5
AR13414	**64**	**128**	**> 128**	**32**	**256**	0.5	**> 64**	**128**	0.5	**> 128**	1	KPC-2, NDM-5
AR13415	**64**	**128**	**> 128**	**32**	**256**	0.25	**> 64**	**128**	0.5	**> 128**	1	KPC-2, NDM-5
AR13416	**128**	**128**	**> 128**	**32**	**256**	0.5	**> 64**	**128**	0.5	**> 128**	0.5	KPC-2, NDM-5
AR13417	**64**	**128**	**> 128**	**32**	**256**	1	**64**	**64**	0.5	**> 128**	1	KPC-2, NDM-5
AR13418	1	**4**	0.5	**2**	8	1	4	2	0.125	**> 128**	**8**	NDM-1
AR13419	**64**	**128**	**> 128**	**32**	**512**	0.5	**> 64**	**128**	0.5	**> 128**	1	KPC-2, NDM-5
AR13420	**64**	**128**	**> 128**	**32**	**256**	0.5	**> 64**	**64**	0.5	**> 128**	1	KPC-2, NDM-5
AR13421	**64**	**128**	**> 128**	**64**	**256**	0.5	**> 64**	**128**	0.5	**> 128**	2	KPC-2, NDM-5
AR13422	**128**	**128**	**> 128**	**32**	32	0.5	**> 64**	**128**	0.5	**> 128**	1	KPC-2, NDM-5
AR13423	**64**	**128**	**> 128**	**32**	**512**	0.5	**> 64**	**128**	0.5	**> 128**	0.5	KPC-2, NDM-5
AR13424	**64**	**256**	**> 128**	**> 64**	64	0.25	**> 64**	**> 128**	0.5	**> 128**	2	KPC-2, NDM-5
AR13425	**64**	**128**	**> 128**	**32**	**512**	1	**> 64**	**128**	0.5	**> 128**	1	KPC-2, NDM-5
AR13427	**32**	**128**	**> 128**	**32**	**1024**	0.5	**> 64**	**64**	0.5	**> 128**	1	KPC-2, NDM-5
AR13428	**128**	**256**	**> 128**	**32**	**1024**	0.5	**> 64**	**> 128**	0.5	**> 128**	1	KPC-2, NDM-5
AR13429	**32**	**128**	**> 128**	**32**	16	0.5	**> 64**	**> 128**	1	**> 128**	1	KPC-2, NDM-5
AR13431	**64**	**128**	**> 128**	**32**	**512**	0.5	**> 64**	**128**	0.5	**> 128**	0.5	KPC-2, NDM-5
AR13443	**64**	**256**	**> 128**	**64**	**512**	0.25	**> 64**	**128**	0.5	**> 128**	1	KPC-2, NDM-5

MIC in bold indicates exceeding the resistance breakpoint

*IMP* imipenem, *MEM* meropenem, *AMK* amikacin, *LEV* levofloxacin, *FOS* fosfomycin, *COL* colistin, *MEV* meropenem/vaborbactam, *IMR* imipenem/relebactam, *AZA* aztreonam/avibactam, *CZA* ceftazidime/avibactam, *TGC* tigecycline, *MIC* minimum inhibitory concentration

### Genomic molecular characteristic

WGS analysis revealed that all AFM-2-PA belonged to sequence type (ST) 275 and KPC-87-PA belonged to ST270 (Figure S1). The other two strains belonged to ST2414 and ST357, respectively. Two separate clusters of closely related (less than 5 allele differences) core genome sequences of 12 clinical CZA-resistant CRPA isolates were identified in a minimum spanning tree analysis (Figure S2). The predominant serotype was O11 (66.7%, 8/12) which is common in the environment and hospital outbreaks, followed by O12 (25%; 3/12) which are more likely to be extensive drug-resistant (XDR). Further analysis showed that antimicrobial resistance gene profiles were very different between AFM-2-PA strains and KPC-87-PA strains (Table S2). KPC-87-PA strains had the same acquired antimicrobial resistance gene profile (Figure S1). Strain-specific gene losses (e.g., *bla*_AFM-2_ in AR13440) further highlighted genomic instability within this cluster (Figure S1).

KPC-2-NDM-5 CRKP strains were classified as ST11-KL47 and the NDM-1 producing CRKP strain belonged to ST29-KL54. All of these CRKP strains carried ESBL genes, the most common being *bla*_CTX-M-65_ (Figure S3). KPC-2-NDM-5 CRKP strains exhibited a conserved resistance gene profile, with AR13409 showing minor variations (e.g., loss of *rmtB* and *fosA3*, gain of *bla*_CTX-M-15_ and *tet(A)*). In Table S2, strain AR13428 had the highest copy numbers for *bla*_NDM_ (5.931) and *bla*_KPC_ (6.983), whereas strain AR13418 had the lowest *bla*_KPC_ copy number (0.033). And the 300 bp upstream sequences of *bla*_NDM_ in all strains were identical.

### Ceftazidime/avibactam resistance mechanism analysis

**Fig. 2:**
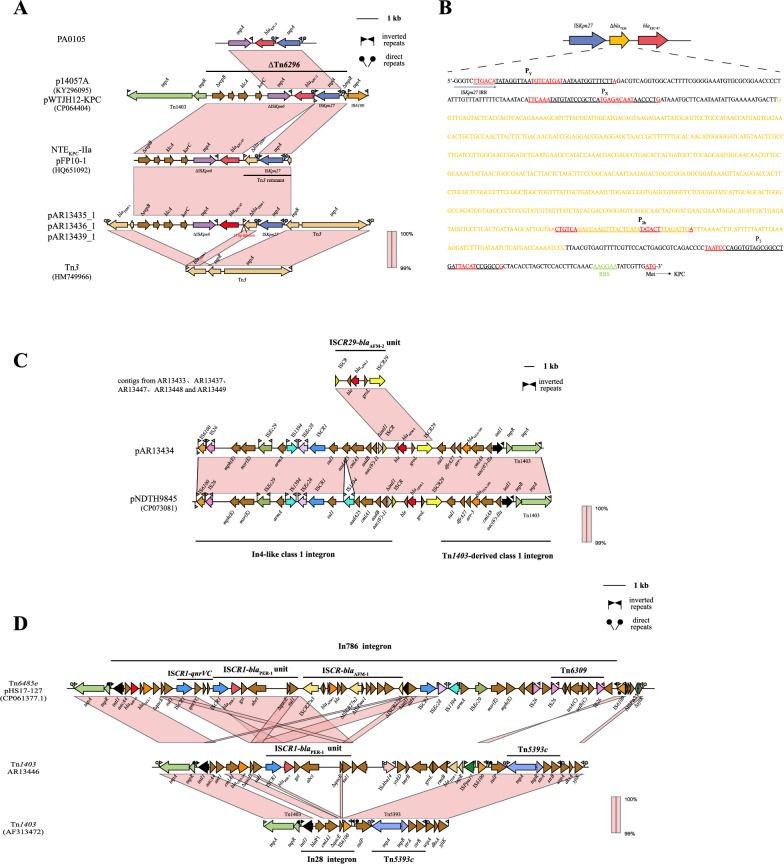
Genetic context analysis of *bla*_KPC-87_, *bla*_AFM-2_ and *bla*_PER-1_. **A** Genetic context comparison of *bla*_KPC-87_ and *bla*_KPC-2_. Shading denotes nucleotide identity > 99%. **B** Promoter region comparison of *bla*_KPC-87_ and *bla*_KPC-2_. Sequence of Δ*bla*_TEM-1_ were labeled in yellow. Putative promoter regions are underlined, in which the − 35 and − 10 and + 1 promoter elements are shown in red characters. RBS, ribosome binding site. **C** Genetic context comparison of *bla*_AFM-2_. Shading denotes nucleotide identity > 99%. **D** Genetic context comparison of *bla*_PER-1_. Shading denotes nucleotide identity > 99%

We further analyzed the resistance mechanism of CRPA and CRKP strains to CZA, especially the high-level resistant strains (MIC > 128 mg/L). The presence of NDM-5, which is not affected by avibactam, is a common cause of resistance to CZA in CRKP strains. We concluded that *bla*_NDM_ carriage is the mechanism of resistance to CZA in 20 CRKP strains of our study. Seven CRPA strains produced AFM-2, which as a MBL, also confers high-level CZA resistance to the host [22].The other three CRPA strains which were resistant to CZA at high level produced KPC-87 which was identified in CZA-resistant *Klebsiella pneumoniae* strains in the previous report [23]. The mutation of KPC-2 to KPC-87 is due to the deletion of three nucleotides of GCA after position 721, resulting in the amino acid substitution of GT241A. Cloning vectors for *bla*_KPC-2_ and *bla*_KPC-87_ with their native promoter were constructed to determine whether *bla*_KPC-87_ mediated CZA resistance. Compared with pGK-KPC2, pGK-KPC87 conferred a 4- to 8-fold MIC increase in CZA, but a variable decrease in the MIC of other β-lactams except for ceftazidime (Table 3). WGS analysis revealed a truncated *bla*_TEM-1_ (Δ*bla*_TEM-1_) existed upstream of *bla*_KPC-87_ which was different from the *bla*_KPC-2_ carrying reference strain (Fig. 2A). Therefore, recombinant *bla*_KPC-2_ and *bla*_KPC-87_ plasmids were constructed with the same promoter sequences (Table 3 and Fig. 2B). With pGK-KPC2-STPT as a reference, pGK-KPC87-STPT increased the MICs of CAZ and CZA but decreased the MICs of meropenem, imipenem, piperacillin, and piperacillin/tazobactam significantly. Specifically, pGK-KPC87-STPT conferred an eightfold MIC increase in CAZ and a 2- to 32-fold MIC increase in CZA (Table 3). Further enzymatic kinetics revealed the effect of GT241A substitution on the hydrolytic activity of KPC-87 (Table S3). Compared with KPC-2, catalytic efficiencies of KPC-87 for nitrocefin and meropenem decreased sharply and even hydrolysis of meropenem was not detected (Figure S4). Nevertheless, the* k*_cat_/*K*_m_ value of KPC-87 for ceftazidime increased by 1.5 times, and *K*_i_ value of KPC-87 for avibactam increased by 7.5 times compared with KPC-2.

**Table 3:** MICs of β-lactams and BLBLI for transformants

Strains	MICs (mg/L)							
	MEM	IMP	PIP	TZP	AZT	FEP	CAZ	CZA
DH5α	< 0.06	0.125	0.5	1	< 0.06	< 0.06	0.125	0.125
DH5α-pGK-PER1	< 0.06	0.25	16	4	> 128	8	> 128	4
DH5α-pGK-KPC2	32	32	> 256	> 256	> 128	128	128	1
DH5α-pGK-KPC2-STPT	1	1	64	32	8	0.5	2	0.125
DH5α-pGK-KPC87-STPT	< 0.06	0.25	8	2	4	0.25	16	4
DH5α-pGK-KPC87	< 0.06	0.25	32	2	8	2	128	16
PAO1	1	0.5	8	8	4	4	2	2
PAO1-pGK-PER1	1	0.5	32	8	> 128	64	> 128	16
PAO1-pGK-KPC2	> 128	> 128	> 256	> 256	> 128	> 128	> 128	4
PAO1-pGK-KPC2-STPT	4	1	4	8	16	32	4	2
PAO1-pGK-KPC87-STPT	1	0.5	8	4	8	16	32	8
PAO1-pGK-KPC87	1	0.5	16	8	16	64	> 128	32

*IMP* imipenem, *MEM* meropenem, *PIP* piperacillin, *TZP* piperacillin/tazobactam, *AZT* aztreonam, *FEP* cefepime, *CAZ* ceftazidime, *CZA* ceftazidime/avibactam, *MIC* minimum inhibitory concentration, *BLBLI* β-lactam–β-lactamase inhibitor combination; *pGK-KPC2* the natural upstream sequences of the KPC-2 gene were retained in the plasmid, including the PY promoter, *pGK-KPC2-STPT/pGK-KPC87-STPT* the PY promoter was removed from the plasmid by PCR and homologous recombination, and only the PX and P1 promoters were retained, *pGK-KPC87* the natural upstream sequences (about 1 kb) of the KPC-87 gene were retained in the plasmid, excluding the PY promoter but retaining the PX and P1 promoters

Notably, *bla*_KPC-87_ did not make DH5α and PAO1 reach above the breakpoint of CZA without Δ*bla*_TEM-1_. The insertion sequence of Δ*bla*_TEM-1_ increased the MICs of *bla*_KPC-87_ transformants for most β-lactam antibiotics we tested, especially for ceftazidime and CZA, and made DH5α and PAO1 resistant to CZA. Besides the promoters PY, PX and P1, bioinformatics analysis revealed a promoter named P2b in Δ*bla*_TEM-1_ with the − 10 and − 35 sequences of TATACT and CTGTCA, respectively (Fig. 2B) [11, 24]. We supposed that the insertion sequence of Δ*bla*_TEM-1_ enhanced the expression of *bla*_KPC-87_ (Table 3). Therefore, we detected the expression level of *bla*_KPC-87_ in the PAO1 transformants and the insertion sequence of Δ*bla*_TEM-1_ increased the expression level of *bla*_KPC-87_ by twofold (Figure S5). The expression level of *bla*_KPC-87_ with insertion sequence of Δ*bla*_TEM-1_ in the transformants was similar to that in clinical isolates. GT241A substitution did not decrease the expression of *bla*_KPC-87_. However, the high expression of KPC-2 caused by Δ*bla*_TEM-1_ did not directly lead to CZA resistance (Figure S6). These results demonstrate that the GT241A substitution in KPC-87, rather than enhanced expression of KPC-2 alone, is essential for conferring CZA resistance. Therefore, GT241A substitution and high expression level caused by the insertion of Δ*bla*_TEM-1_ synergistically mediated the CZA resistance of clinical isolates.

It is worth noting that AR13446, although not carried the carbapenemase genes, carried the inhibitor-resistant extended-spectrum beta-lactamase (ESBL) gene *bla*_PER-1_ which mediates CZA resistance.

Cloning experiments showed that *bla*_PER-1_ increased the MIC of CZA in PAO1 to 16 mg/L, even though it only increased the MIC of CZA in DH5α to 4 mg/L (Table 3). We further investigated the contribution of efflux pumps to CZA resistance. With the presence of PAβN, strains carrying *bla*_KPC-87_ and *bla*_AFM-2_ still exhibit high-level resistance to CZA, but other strains showed a significant decrease (by 8–64 fold) in MIC and became sensitive to CZA.

All KPC-87-PA strains harbored frameshift mutations in *oprD*, deemed nonfunctional, likely contributing to imipenem resistance despite KPC-87 reduced carbapenemase activity. In accordance with previous reports, efflux pump hyperactivity MexAB-OprM actively extrudes meropenem, but not imipenem[25].

### Genetic context of *bla*_KPC-87_,* bla*_AFM-2_ and *bla*_PER-1_

KPC-87-PA strains (AR13435, AR13436, and AR13439) had the same genetic context of *bla*_KPC-87_. The core genetic element IS*Kpn27*-*bla*_KPC_-ΔIS*Kpn6* remained high similarity in the studied clinical isolates and reference strains (Fig. 2A). In the studied clinical isolates, the genetic structure of *bla*_KPC-87_ was a transposon based on Tn*3* backbone. Two *bla*_TEM-1_ sequences were detected in this transposon structure and left inverted repeats (IRL) were found downstream of both *bla*_TEM-1_ sequences, but 5-bp direct repeats (DR), GACTA, were merely found downstream of the complete *bla*_TEM-1_. Δ*bla*_TEM-1_ was identified between IS*Kpn27* and *bla*_KPC-87_. According to previous studies, this Tn*3*-like transposon carrying *bla*_KPC-87_ was classified as type II *bla*_KPC_ carrying non-Tn*4401* elements (NTE_KPC_-II), which was similar to NTE_KPC_-IIa. Sequence alignment with NTE_KPC_-IIa showed that Δ*bla*_TEM-1_ were consistent and a 15 bp deletion occurred between Δ*bla*_TEM-1_ and *bla*_KPC_ in our strains. Compared with *bla*_KPC_ region of type I and type II plasmid, NTE_KPC_-IIa-like element contained ΔTn*6296*-like sequences and its Tn*3* remnant contained additional Δ*bla*_TEM-1_.

IS*CR29*-*bla*_AFM-2_ unit was detected from contigs of all *bla*_AFM-2_ carrying strains (Fig. 2C). The insertion of IS*CR29*- *bla*_AFM-2_ unit resulted in the truncation of integrons on both sides. The partial downstream of the left In4-like integron had been replaced by *armA* and *msr(E)-mph(E)* as described previously. The 5′-conserved segment (5′-CS) of the In4-like integron and the 3′-CS of Tn*1403*-derived integron were truncated by the inserted sequence. The direct repeats (GGTTT) and inverted repeat IRt of In4-like integron were merely found downstream of IS*6100* (Fig. 2C). The right integron, like Tn*1403*-derived class 1 integron in pNDTH9845, carried cassette array *aac(6*′*)-IIa-cmlA8-bla*_OXA246_*-arr-3-dfrA27*. The inverted repeat IRt of Tn*1403*-derived integron was lost. The mapping of genomic reads to the genetic context of *bla*_AFM-2_ showed that only AR13437 and AR13440 could not completely cover the region, and they missed *armA*, *msr (E)*, *mph(E)* and some IS sequences (Figure S7A). AR13440 even lacked IS*CR29*-*bla*_AFM-2_ unit.

The transposon in which *bla*_PER-1_ was located originated from Tn*1403* (Fig. 2D). The segment IS*CR1*-*bla*_PER-1_-*gst-abct-ΔqacE-sul1* could form a transposable cycling molecule. The internal resistance genes of integron in pAR13446 had changed greatly but its main structures including *intI1*, IS*6100*, IR, and DR were exactly identical to In28. And downstream sequence of In28-like integron containing Tn*5393c* was identical to Tn*1403*. Compared with *ΔqacE* in 3′-CS of In28, a 79-bp deletion was found in Δ*qacE* downstream *bla*_PER-1_. The In28-like integron carried cassette array *accA4-ant1-cmlA1-bla*_OXA-246_. The downstream sequence of IS*CR1*-*bla*_PER-1_ unit in In28-like integron also carried *yokD*, *tmrB,* and *rmtB*, as well as Tn*3* remnant. The same IS*CR1*-*bla*_PER-1_ unit was inserted in the In783 intergron.

The plasmids containing *bla*_KPC-87_ in KPC-87-PA strains (pAR13435_1, pAR13436_1, and pAR13439_1) were entirely identical and the plasmids size were 452,253 bp. The contigs of ST275 strains were mapped to the plasmid pAR13434_1 and the mapping coverage was between 61.2 and 82% (Figure S7B). S1-PFGE revealed that ST275 harbored plasmids of varying sizes, ranging from 336.5 to 480 kb (Figure S7C).

### Transmission of CZA-resistant CRPA and CRKP

KPC-2-NDM-5 CRKP strains were consistently isolated from ICU patients (Fig. 1A), so we further calculated the SNP differences among KPC-2-NDM-5 CRKP strains. The heatmap indicated that KPC-2-NDM-5 CRKP could be classified into three clusters based on SNP differences (Figure S8). The SNP differences within the three clusters were 0–20, 1–29 and 13–52, respectively. CRKP Cluster 1 strains were successively isolated from patient P14 during November–December 2020 (Fig. 1A). It is suggested that cluster1 represented in vivo microevolution of KPC-2-NDM-5 CRKP strains. However, it is notable that the strains in cluster 2 and cluster 3 were isolated from different patients, and there was a time span between the isolations. For strains with SNP < 25 in cluster 2, the isolation interval ranged from 1–77 days, while for strains with SNP < 25 in cluster 3, the isolation interval ranged from 42–150 days. It was hypothesized that two distinct KPC-2-NDM-5 CRKP clones diffused in the ICU, forming the cluster 2 and cluster 3. Patient P14 was associated with strains from all three clusters of KPC-2-NDM-5 CRKP and may be an intermediate in the transmission of cluster 2 and cluster 3.

We found two main clusters, ST270 cluster and ST275 cluster, in 12 clinical CZA-resistant isolates. Within two clusters, no allelic differences between AR13433 and AR13437 and between AR13435 and AR13436, and each pair was isolated from the same patient (P8 and P20, respectively). However, there were no allelic differences between AR13434 and AR13447 isolated from different patients (P3 and P6). ST275 cluster presented a special situation that strains exhibited narrow cgMLST-based allelic differences alongside wide SNP variations (Figure S9A). ST275 cluster can further be divided into two subclusters (Figure S9B). The strains in cluster 1 were isolated from four different patients while the strains in cluster 2 were isolated from the same patient P8 (Fig. 1A). The SNP difference analysis of the core genome of ST275 strains showed that even for cluster 2, the core genome SNP differences varied from 188 to 1056 SNPs. The SNPs of cluster 1 varied from 228 to 538 SNPs. Otherwise, we found that the strains from cluster 1 could fully map the genetic context of *bla*_AFM-2_ in AR13434 by mapping analysis between ST275 strains and the genetic context of *bla*_AFM-2_. The strains from cluster 2, except for AR13433, had partial deletions in the genetic context of *bla*_AFM-2_, while AR13440 even lacked the IS*CR29*- *bla*_AFM-2_ unit (Figure S7A). The most important thing was that the size of plasmids in ST275 strains was irregular (Figure S7C). ST275 strains carried contigs that mapped to the plasmid pAR13434_1, with mapping coverage ranging from 61.2 to 82%. According to the results of S1-PFGE and plasmid mapping, we supposed that the backbones of plasmids carried by ST275 strains were similar to pAR13434_1. In conclusion, we believed that the ST275 strains had disseminated and undergone varying degrees of evolution within the hospital setting.

**Fig. 3:**
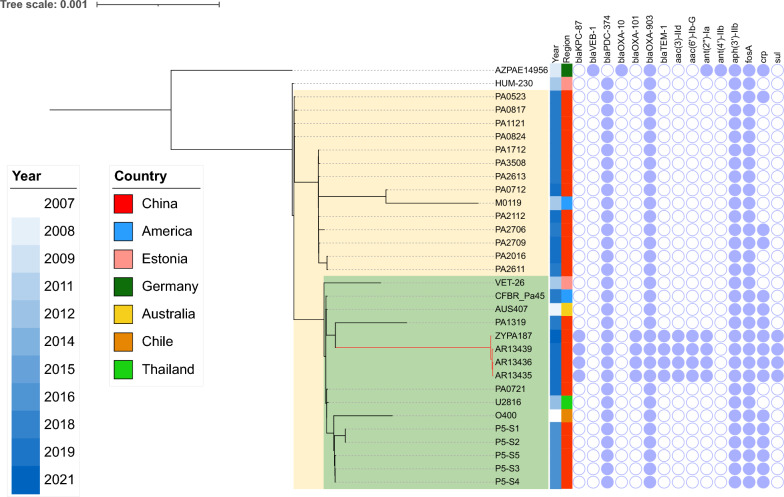
The phylogenetic tree of ST270 *P. aeruginosa* strains. Core-genome phylogenetic tree was built for 32 ST270 *P. aeruginosa* strains and rooted in the midpoint. Isolation year was indicted by different shades of blue. The clade 1 was labeled in yellow shade and the clade 2 was labeled in green shade. The red branches are the branches where the ST270 *P. aeruginosa* strains in this study. Scale bar indicates nucleotide substitutions per site

The difference in SNPs of the core genome in ST270 cluster was between 9 and 24 SNPs (Fig. 3A). Moreover, AR13435 and AR13436 were isolated from the same patient and the SNPs difference was 9 SNPs, while the SNPs difference between AR13435 and AR13439 was 23 SNPs. Since the ST270 strains carried an exactly identical *bla*_KPC-87_ bearing plasmid and had little difference in alleles and SNPs, we speculated that ST270 strains transmitted from patient P20 to patient P24.

We were interested in ST270 *P. aeruginosa*, so we constructed a phylogenetic tree of ST270 strains from NCBI and our database. Almost ST270 strains were isolated in China in 2017–2021(Fig. 3C). ZYPA187, which located on the same branch as AR13435, AR13436 and AR13439, was isolated from Zhejiang, China. ZYPA187 carried the same resistance gene profile as other strains on the same branch, and they carried more resistance genes than other ST270 strains. Even the pZYPA187 (accession no. CP133754) had a high similarity with pAR13435_1 (99% coverage, 100% identity). The difference in SNP between ZYPA187 and the other strains on the same branch amounted to 91–101 SNPs. We traced the source of ZYPA187 and discovered that ZYPA187 was isolated from patient P17 in our study after the referral, and AFM-2-PA strain AR13449 was also previously isolated from the P17 patient. The isolation time of AR13449 and ZYPA187 was only 18 days apart. We speculated that ZYPA187 had already transmitted to patient P17 before the referral, caused bloodstream infection and was subsequently isolated after the referral.

## Discussion

*bla*_KPC_ is the most common carbapenemase gene in CRPA and CRKP, especially found in China[11, 26–28]. The prevalence of *bla*_KPC-2_ in CRPA ranges from 11.9 to 92.1%, while it can be as high as 79.5–100% in CRKP [11, 13, 26–29]. The most common mechanism of resistance to CZA is the presence of class B and some class D β-lactamases [3]. In the present study, almost all CRKP strains carried both KPC-2 and NDM-5, whereas most CRPA strains carried AFM-2, suggesting to some extent that access to the MBL is more likely to achieve CZA resistance in both CRKP and CRPA strains. CRKP strains can improve fitness by reducing NDM expression, which may explain the low level of carbapenem resistance in AR13418 [30, 31].

KPC-87 is a novel KPC variant that exhibits stronger avibactam resistance (7.5-fold higher* K*_i_ value), enhanced ceftazidime hydrolytic activity (1.5-fold higher *k*_cat_/*K*_m_), and reduced carbapenemase activity compared to KPC-2. Furthermore, our expanded genomic and mechanistic analyses revealed a multifactorial resistance landscape, consistent with established mechanisms in *K. pneumoniae*, *P. aeruginosa* and other Gram-negative pathogens. Its emergence aligns with global trends of KPC diversification under CZA selective pressure. KPC mutants conferring CZA resistance show significant changes in enzyme kinetic parameters to three drugs: ceftazidime, carbapenems and avibactam. For instance, KPC-41, identified in Switzerland, similarly exhibit elevated avibactam resistance (fourfold higher* K*_i_) while retaining partial carbapenemase activity [32]. KPC-87 lose carbapenem hydrolysis, like KPC-33 in Greece [33, 34], reflecting global convergent adaptation to CZA pressure in high-usage regions [4]. But KPC-87 has a significantly higher hydrolysis of ceftazidime, its dual mechanism—heightened avibactam resistance and substrate-specific catalysis—differentiates it from other variants, elevating risks of evading therapies and diagnostics. Moreover, we have examined whether increased expression of KPC-2 alone (driven by the native promoter of KPC-87) could confer resistance to CZA. PAO1 transformants expressing KPC-2 under the native promoter of KPC-87 (without the GT241A mutation) and found that the PY promoter, as a strong promoter, significantly increased the expression level of *bla*_KPC-2_, which led to an increase in the MIC value of the pGK-KPC2 strain against CAZ. In contrast, in the pGK-KPC2-STPT strain, the expression level of *bla*_KPC-2_ was reduced due to the deletion of the PY promoter, resulting in a corresponding decrease in the MIC value. This result is consistent with the direct correlation of promoter strength on β-lactamase expression and drug resistance phenotype.

Indeed, many studies of KPC variants have ignored the contribution of promoters to CZA resistance. Tn*4401* variants were identified as the classical genetic structure surrounding *bla*_KPC_ but NTE_KPC_ was more common in *bla*_KPC_-harboring *P. aeruginosa* strains [29]. The genetic structure of *bla*_KPC-87_ in our study was classified as NTE_KPC_-II due to the presence of Δ*bla*_TEM_ between IS*Kpn27* and *bla*_KPC_. The insertion of 656 bp Δ*bla*_TEM_ enhanced the *bla*_KPC-87_ expression significantly. Importantly, the hyperexpression of *bla*_KPC-87_ driven by the promoter variant of Δ*bla*_TEM-1_ highlights a novel evolutionary strategy where cis-regulatory mutations amplify resistance phenotypes without requiring gene duplication. Such findings underscore the need for surveillance programs targeting promoter regions in *bla*_KPC_-harboring strains, as current resistance screening often overlooks these regulatory elements.

Infections caused by some CZA-resistant KPC variant-producing *Enterobacterales* strains can be rescued (clinically resolved) with meropenem or meropenem/vaborbactam [35–37]. However, due to the complex drug resistance mechanism, *P. aeruginosa* may remain resistant to carbapenems following KPC mutation. Furthermore, KPC-87-PA was resistant to several kinds of BLBLI (meropenem/vaborbactam, imipenem/relebactam, and aztreonam/avibactam) in our study. All KPC-87-PA strains harbor frameshift mutations in oprD, deemed nonfunctional, likely contributing to imipenem resistance despite KPC-87 reduced carbapenemase activity [25]. Prior studies report comparable MICs for meropenem/vaborbactam and meropenem alone, indicating that vaborbactam fails to mitigate co-existing non-enzymatic resistance mechanisms [38, 39].The emergence of resistance to BLBLI combinations severely limits the range of available antimicrobial agents for treating patients infected with KPC-87-PA. In our study, the only available antimicrobials for KPC-87-PA were amikacin, fosfomycin and colistin. Previous studies often reported CZA-resistant strains from patients during or after CZA treatment [40]. The first KPC-87-PA in our study was isolated from patient P20 before CZA treatment. The AFM-2-PA was an epidemic clone resistant to CZA in this hospital according to whole genome analysis. The KPC-87-PA had a small range of dissemination but spread across regions with the referral of a patient. The transmission dynamics of CZA-resistant strains within the ICU (ST11 CRKP clusters) and beyond (ST270 CRPA via patient referral) highlight weaknesses in current infection control frameworks. The identification of ST270 CRPA in geographically distinct hospitals (like Zhejiang and Jiangsu) signals a broader regional threat. Previous studies reported nosocomial transmission of ST773 CRPA within and between the three major hospitals in Fiji and environmental-to-patient transmission of ST235 CRPA carrying *bla*_IMP-84_ in Idaho [41, 42]. The rise of MBLs-producing *K. pneumoniae* in ICUs in Argentina and Greece has been linked to CZA use, suggesting a similar trend for NDM-5 and AFM-2 in our study [43, 44]. To curb environmental spread of resistant bacteria, implement enhanced disinfection protocols, genomic surveillance networks for cross-institutional tracking, and targeted interventions like pre-ICU decolonization.

While our study provides valuable insights into the mechanisms and transmission dynamics of CZA-resistant CRKP and CRPA, several limitations should be acknowledged. First, the small sample size (32 CZA-resistant strains from a single ICU) limits generalizability, particularly for novel mechanisms like KPC-87 and AFM-2. Second, the regional focus on isolates from eastern China introduces potential geographic bias. Third, we identified environmental transmission as a potential risk but lacked systematic environmental or healthcare worker sampling. Future work should expand multicenter collaborations to assess geographic prevalence, integrate genomic-epidemiological surveillance to track resistance evolution, and conduct environmental metagenomics to clarify pathogen reservoirs in outbreaks.

## Supplementary Information

Additional file1 (PDF 4125 KB)

## Data Availability

The sequence data of CRPA strains and CRKP strains were deposited in the DDBJ/ENA/GenBank database under BioProject accession number PRJNA1049648 and PRJNA1090252, respectively. The sequence data of ST270 P. aeruginosa strains and PA0105 used in clone experiment were deposited in the n the DDBJ/ENA/GenBank database under BioProject accession number PRJNA962265, the accesion number for each sequence is detailed in Table S4.

## Declarations

### Transparency declarations

None to declare.
