# Supplementary material for: Diverse modes of ceftazidime/avibactam resistance acquisition in carbapenem-resistant Klebsiella pneumoniae and Pseudomonas aeruginosa from a Chinese intensive care unit
Source: Ann Clin Microbiol Antimicrob. 2025 May 30;24:35. doi: 10.1186/s12941-025-00800-z (PMC12125794; doi:10.1186/s12941-025-00800-z)
Supplement: Supplementary file 1 — Additional file1 (PDF 4125 KB) [file 12941_2025_800_MOESM1_ESM.pdf]

Supplementary Information

Supplementary Tables

Table S1. Prime used in this study.

Name	Description	Prime sequence (5’ → 3’)
pGK-F/R	For amplification of pGK-1900 vector backbone	F：TTCTCATGGCTCTGCCCTC<br>R：TTCGCCCTATAGTGAGTCGT
KPC_P_F/R	For the construction of pGK-KPC2	F: ACGACTCACTATAGGGCGAACGGTCACG CGCGAGTTCATCAATC<br>R: GAGGGCAGAGCCATGAGAATTACTGCCC GTTGACGCCCAATC
KPC87-F/R	For the construction of pGK-KPC87 and pGK-KPC2-STPT	F: TACGACTCACTATAGGGCGAAGTTAATGT CATGATAATAAT<br>R: GAGGGCAGAGCCATGAGAAGGTGGTGG GCCAATAGATGA
STPT-KPC87-F/R	For the construction of pGK-KPC87-STPT with KPC87-F/R	F: TCAAACAAGGAATATCGTTGATGTCACT GTATCGCCGTCT<br>R: CAACGATATTCCTTGTTTGA
Promoter-KPC-F/R	For the construction of pGK-KPC2-STPT and PAO1-pGK-ΔTEM-KPC2	F: TCAAACAAGGAATATCGTTGATGTCACTG TATCGCCGTCT<br>R: CAACGATATTCCTTGTTTGAAGGTG
pET-F/R	For amplification of pET-28a vector backbone	F: CATATGTCCCTGGAAGTAGAGATTCTC<br>R: GATCCGGCTGCTAACAAAGC
KPC-pET-F/R	For amplification of KPC-2 and KPC-87	F: TCTACTTCCAGGGACATATGCTGACCAAC CTCGTCGCG<br>R: GCTTTGTTAGCAGCCGGATCTTACTGCCC GTTGACGCC
q-rpoD-F/R	For quantitative PCR	F: CTTACGCGGAGGTCAACGAC<br>R: TCCGGGGCTGTCTCGAATAC
q-KPC-F/R	For quantitative PCR	F: CGCCGTCTAGTTCTGCTGTC<br>R: CCGCCAAAGTCCTGTTCGAG

Table S2. Antimicrobial resistance genes of KPC-87-PA, AFM-2-PA, KPC-2-NDM-5 CRKP and NDM-1 CRKP.

Strain	Antimicrobial resistance genes											
	β-lactams	Aminoglycoside	Quinolone	Macrolide	Chloromycetin	Trimethoprim	Bleomycin	Fosfomycin	Rifamycin	Streptomycin	Sulfonamide	Tetracycline
**KPC-87-PA**	*bla*$_{KPC-87}$, *bla*$_{OXA-903}$, *bla*$_{OXA-101}$*, *bla*$_{TEM-1}$	*aac(3)-IId*, *aac(6')-Ib-G*	*crpP**	/	*catB7*	/	/	*fosA*	/	*aph(6)-Id*, *aph(3'')-Ib*	*sul1*	/
**AFM-2-PA**	*bla*$_{AFM-2}$, *bla*$_{OXA-246}$, *bla*$_{OXA-50}$	*armA*, *aph(3')-IIb*, *ant(2'')-Ia*, *aac(6')-Il*, *aac(6')-IIa*	*crpP*, *qnrVC1*	*msr(E)*, *mph(E)*	*cmlA1*, *cmlA8*	*dfrA22*, *dfrA27*	*ble*$_{MBL}$	*fosA*	*arr-3*	*aadA25*	*sul1*	/
**KPC-2-NDM-5 CRKP**	*bla*$_{KPC-2}$, *bla*$_{NDM-5}$, *bla*$_{CTX-M-65}$, *bla*$_{CTX-M-15}$, *bla*$_{OXA-1}$, *bla*$_{SHV-11}$, *bla*$_{TEM-1}$	*aph(3')-Ia*, *aac(6')-Ib-D181Y*, *rmtB1*	*oqxA*, *oqxB*,	/	/	*dfrA12*	*ble*$_{MBL}$	*fosA3*, *fosA6*	/	*aadA2*	*sul1*	*tet(A)*
**NDM-1 CRKP**	*bla*$_{NDM-1}$, *bla*$_{CTX-M-3}$, *bla*$_{SHV-187}$, *bla*$_{TEM-1}$	*aac(6')-Ib-D181Y*	*oqxA10*, *oqxB25*, *qnrS1*	*mph(A)*	*floR*	*dfrA27*	*ble*$_{MBL}$	*fosA6*	*arr-3*	*aadA16*	*sul1*	*tet(A)*

Notes: *, indicates the presence of two identical genes;/, indicates the absence of such resistance genes.

Table S3. Carbapenemase genes copy number analysis for CRKP strains.

Strain	Depth of *bla*$_{NDM}$	Depth of *bla*$_{KPC}$	the average depth of seven MLST alleles	*bla*$_{NDM}$ copy number	*bla*$_{KPC}$ copy number
AR13408	421.087	438.037	141.990	2.966	3.085
AR13409	501.484	550.322	181.595	2.762	3.030
AR13413	468.099	517.503	162.211	2.886	3.190
AR13414	480.674	568.095	176.918	2.717	3.211
AR13415	668.947	773.235	260.842	2.565	2.964
AR13416	439.960	469.995	162.541	2.707	2.892
AR13417	473.548	573.269	181.539	2.609	3.158
AR13419	529.239	590.709	189.206	2.797	3.122
AR13420	630.234	683.509	244.156	2.581	2.799
AR13421	756.516	860.595	274.988	2.751	3.130
AR13422	648.185	673.054	247.405	2.620	2.720
AR13423	355.830	417.372	125.400	2.838	3.328
AR13424	475.205	506.465	186.212	2.552	2.720
AR13425	492.968	598.924	183.523	2.686	3.263
AR13427	486.324	575.126	186.650	2.606	3.081
AR13428	1221.143	1437.757	205.882	5.931	6.983
AR13429	463.681	613.900	243.184	1.907	2.524
AR13431	603.015	720.056	199.212	3.027	3.615
AR13443	364.477	419.207	132.644	2.748	3.160
AR13418	387.282	6.218	187.110	2.070	0.033

Table S4. Kinetic parameters of KPC-2 and KPC-87.

	KPC-2			KPC-87		
	K_m or K_i (μM) [a]	k_{cat} (s^{-1})	k_{cat}/K_m ($\mu M^{-1}s^{-1}$)	K_m or K_i (μM) [a]	k_{cat} (s^{-1})	k_{cat}/K_m ($\mu M^{-1}s^{-1}$)
**NCF**	74.57±5.85	751.43 ±60.65	10.08±0.05	65.71±2.90	41.70±1.92	0.635±0.005
**CAZ**	261.82±19.79	2.47±0.18	（9.45±0.14）$\times 10^{-3}$	38.42±0.83	0.565±0.018	（14.69±0.21）$\times 10^{-3}$
**AVI**	2.30±0.05	/	/	17.27 ±0.42	/	/

K_m, k_{cat} and K_i values are shown as means ± standard deviation from three independent experiments.

NM, not measurable due to a low initial rate of hydrolysis.

NCF, nitrocefin; CAZ, ceftazidime; AVI, avibactam.

[a] The K_m values for NCF and CAZ were displayed and the K_i value for AVI was displayed.

Table S5. Accession number of CRPA and CRKP from ICU in this study (n=32).

Strain ID	Patient ID	MLST	GenBank Accession number	Species
AR13432	P12	ST 2414	SAMN38704713	PA
AR13433	P8	ST 275	SAMN38704714	PA
AR13434	P3	ST 275	SAMN38704715	PA
AR13435	P20	ST 270	SAMN38704598	PA
AR13436	P20	ST 270	SAMN38704599	PA
AR13437	P8	ST 275	SAMN38704716	PA
AR13439	P24	ST 270	SAMN38704600	PA
AR13440	P8	ST 275	SAMN38704717	PA
AR13446	P25	ST 357	SAMN38704718	PA
AR13447	P6	ST 275	SAMN38704719	PA
AR13448	P19	ST 275	SAMN38704720	PA
AR13449	P17	ST 275	SAMN38704721	PA
AR13408	P5	ST 11	SAMN40560478	KP
AR13409	P22	ST 11	SAMN40560479	KP
AR13413	P7	ST 11	SAMN40560480	KP
AR13414	P1	ST 11	SAMN40560481	KP
AR13415	P15	ST 11	SAMN40560482	KP
AR13416	P24	ST 11	SAMN40560483	KP
AR13417	P4	ST 11	SAMN40560484	KP
AR13418	P18	ST 29	SAMN40560485	KP
AR13419	P14	ST 11	SAMN40560486	KP
AR13420	P14	ST 11	SAMN40560487	KP
AR13421	P13	ST 11	SAMN40560488	KP
AR13422	P14	ST 11	SAMN40560489	KP
AR13423	P14	ST 11	SAMN40560490	KP
AR13424	P9	ST 11	SAMN40560491	KP
AR13425	P16	ST 11	SAMN40560492	KP
AR13427	P21	ST 11	SAMN40560493	KP
AR13428	P14	ST 11	SAMN40560494	KP
AR13429	P14	ST 11	SAMN40560495	KP
AR13431	P14	ST 11	SAMN40560496	KP
AR13443	P27	ST 11	SAMN40560497	KP

Notes: PA, *Pseudomonas aeruginosa*; KP, *Klebsiella pnenmoniae*.

Table S5. Accession number of ST270 *P. aeruginosa* strains and PA0105.

Strain	Accession number
PA3508	JBCDWM000000000
PA2709	JBCDWN000000000
PA2706	JBCDWO000000000
PA2613	JBCDWP000000000
PA2611	JBCDWQ000000000
PA2112	JBCDWR000000000
PA2016	JBCDWS000000000
PA1712	JBCDWT000000000
PA1319	JBCDWU000000000
PA1121	JBCDWV000000000
PA0824	JBCDWW000000000
PA0817	JBCDWX000000000
PA0721	JBCDWY000000000
PA0712	JBCDWZ000000000
PA0523	JBCDXA000000000
PA0105	JBCDXB000000000

Supplementary Figures

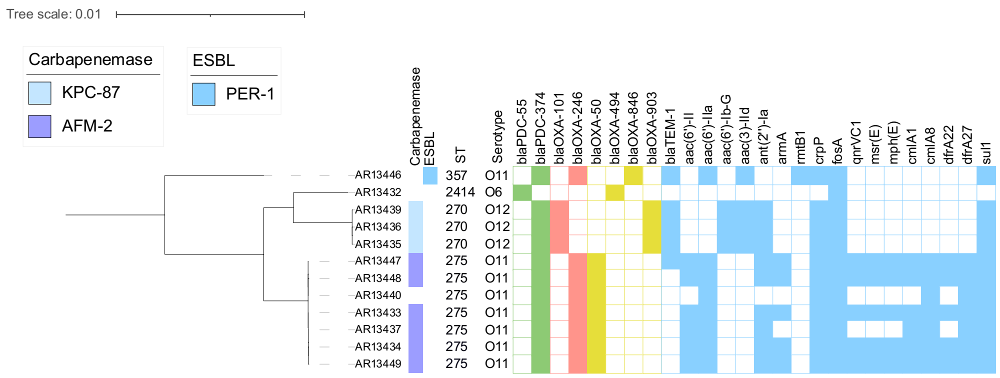

Figure S1. The phylogenetic tree of 12 CZA-resistant *P. aeruginosa* strains.
Green boxes indicate *ampC*, red boxes indicate *bla*$_{\mathrm{OXA\text{-}10}}$ like genes, yellow boxes indicate *bla*$_{\mathrm{OXA\text{-}50}}$ like genes. Scale bar indicates nucleotide substitutions per site.

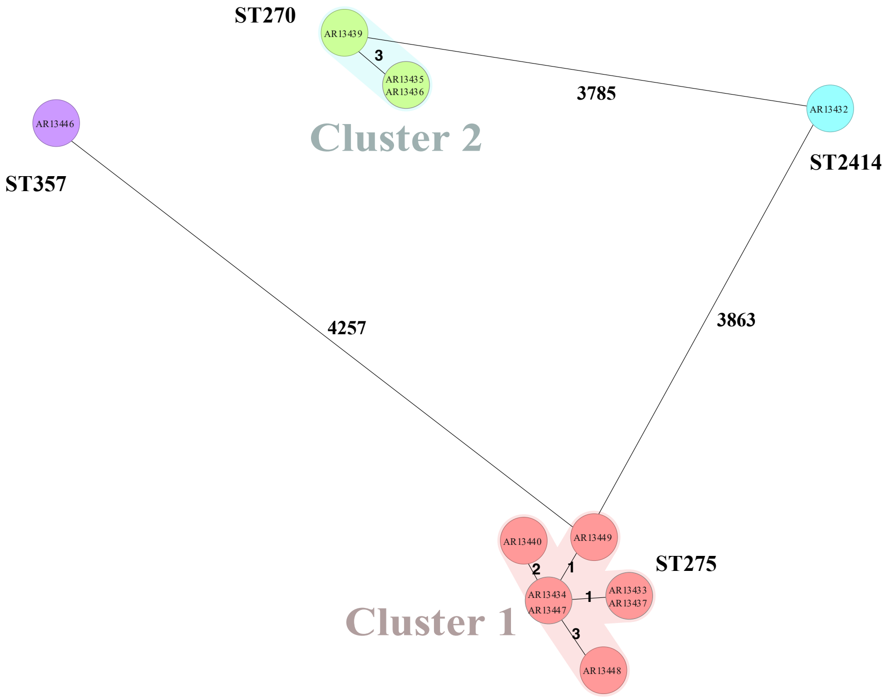

Figure S2. cgMLST-based minimum spanning tree of 12 CZA-resistant *P. aeruginosa* strains. The strain name is represented in the circle.

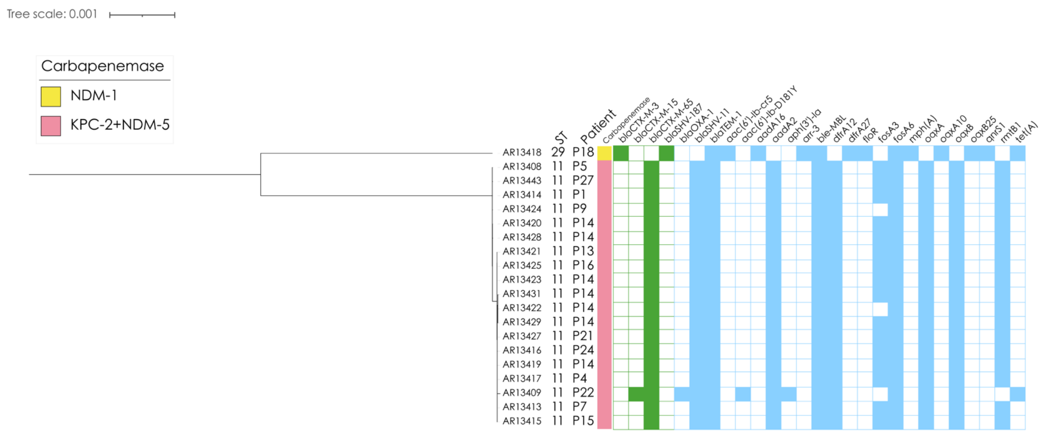

Figure S3. Phylogenetic tree of *Klebsiella pneumoniae* strains

Coloured squares represent the presence of genes and blank squares represent the absence of genes. The green squares represent the extended spectrum β-lactamase genes and the blue squares represent other acquired antibiotic resistance genes.

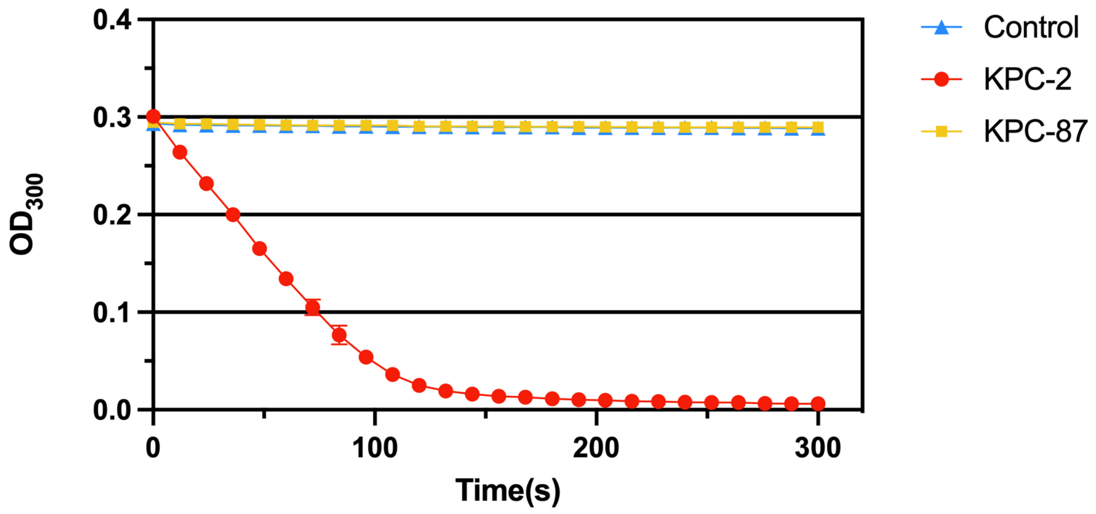

Figure S4. Progress curves of KPC-2 (red), KPC-87 (yellow) and no enzyme control (blue) for meropenem hydrolysis. Progress curves of KPC variants for meropenem hydrolysis were generated by measuring absorbance at 300 nm with 30 nM enzymes and 50 µM meropenem.

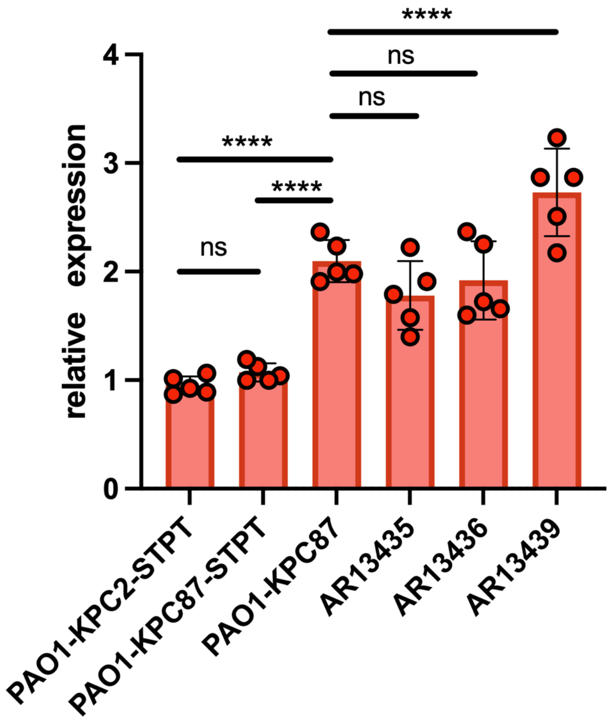

Figure S5. Expression level of KPC in transformants and clinical isolates.

Each individual point represents a monoclonal; The bars represent the mean and standard error of the mean (mean±SD); ns, no significant difference; ****, *P*<0.0001.

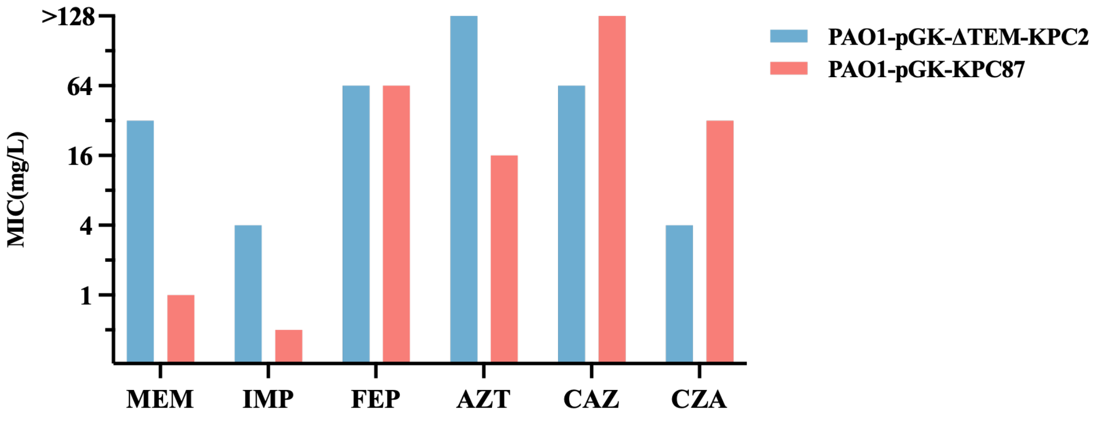

Figure S6. MICs of PAO1 transformants with KPC-2 and KPC-87.

IMP, imipenem; MEM, meropenem; AZT, aztreonam; FEP, cefepime; CAZ, ceftazidime; CZA, ceftazidime/avibactam; MIC, minimum inhibitory concentration. PAO1-pGK-ΔTEM-KPC2 and PAO1-pGK-KPC87 had the same native promoter of KPC-87.

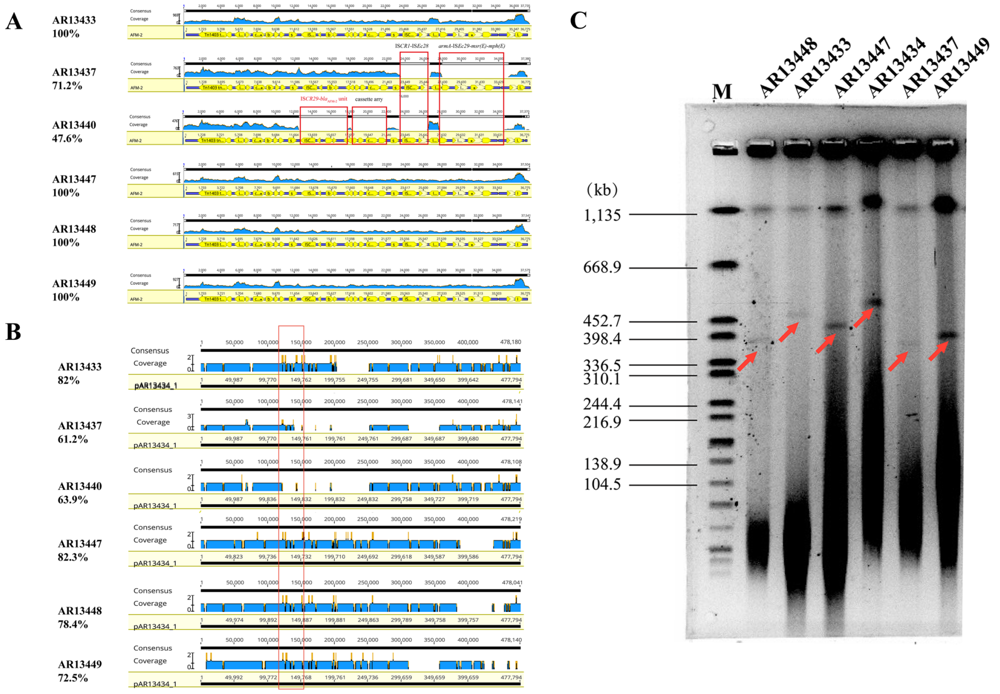

Figure S7. Plasmid comparison analysis of AFM-2 bearing plasmids.
(A)Mapping of the genetic context of AFM-2 by ST270 strains. Red boxes indicated missing regions in the genetic context of AFM-2. (B)Mapping of pAR13434_1 by ST270 strains. Rend box indicated the genetic context of AFM-2. (C)S1-PFGE of AFM-2 carrying strains. M, *Salmonella Braenderup* H9812 digested by *Xba*I for marker. Red arrows indicated plasmids of AFM-2 carrying strains.

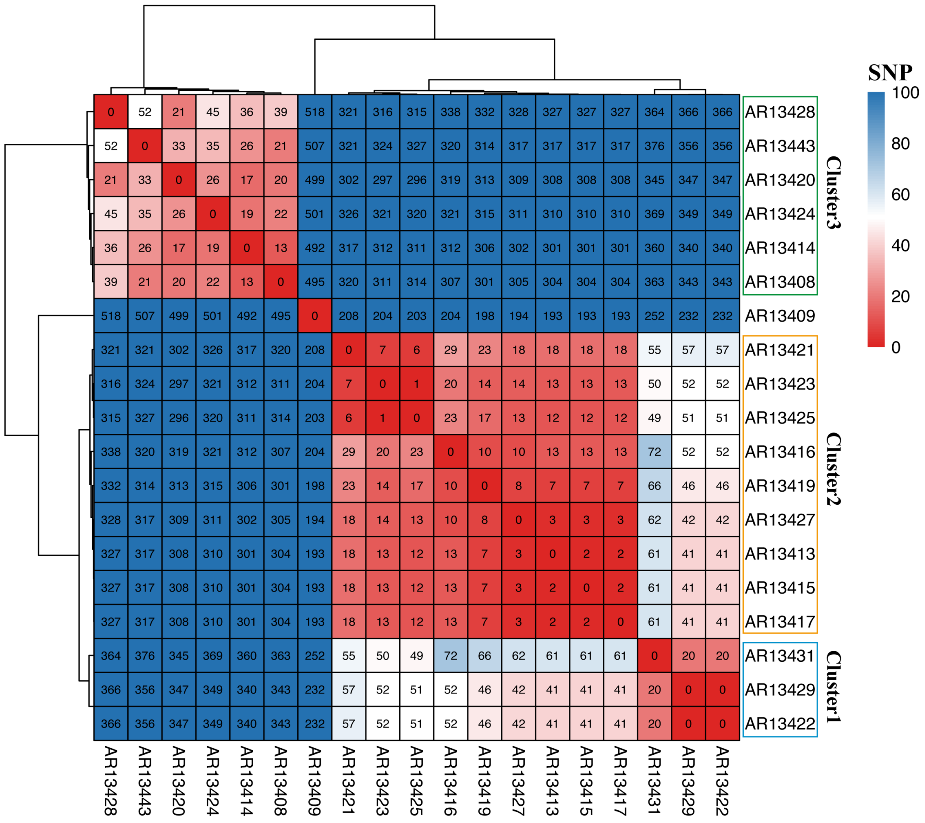

Figure S8. Heatmap of Klebsiella pneumoniae core genome SNP.

The strains framed by blue line are cluster 1, the strains framed by orange line are cluster 2 and the strains framed by green line are cluster 3.

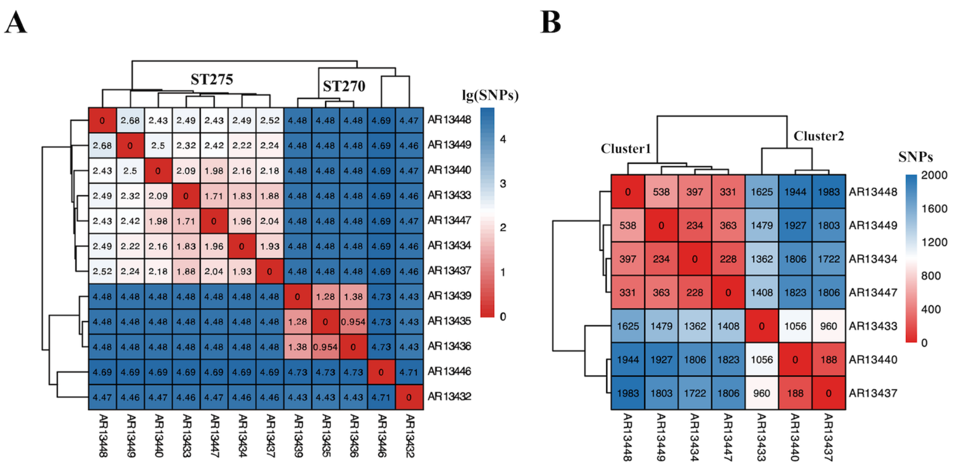

Figure S9. Heat map of SNPs in 12 CZA-resistant *P. aeruginosa* and the phylogenetic analysis of ST270 *P. aeruginosa* strains. (A) Heat map of SNPs in 12 CZA-resistant *P. aeruginosa*. SNPs are displayed with lg (SNPs) based on core genome of 12 CZA-resistant *P. aeruginosa* strains. (B) Heat map of SNPs among 7 ST275 *P. aeruginosa* strains.
